# webSalvador: a Web Tool for the Luria-Delbrük Experiment

**DOI:** 10.1128/MRA.00314-21

**Published:** 2021-05-20

**Authors:** Qi Zheng

**Affiliations:** a Department of Epidemiology and Biostatistics, Texas A&M University School of Public Health, College Station, Texas, USA; Indiana University, Bloomington

## Abstract

Existing Web tools for the Luria-Delbrück fluctuation experiment do not offer many desirable capabilities that are vital to mutation research. webSalvador offers these capabilities via a user interface that allows researchers to access most of the functions in the R package rSalvador without having to learn the R language.

## ANNOUNCEMENT

For nearly 8 decades the Luria-Delbruck protocol (1) has been the principal tool for determining microbial mutation rates in the laboratory. In 2009, the Web tool FALCOR (2) acted as a spur to applications of the classic protocol. The second Web tool, bz-rates (3), added a feature to adjust for plating efficiency. The third Web tool, FluCalc, offered an improved user interface (4). The present Web tool, webSalvador, is intended to fill several gaps left by the existing Web tools. Some of these gaps are highlighted by the following data: 22 16 44 39 26 36 35 19 26 25 35 33.

I first regard the data as mutant counts from the entirety of 12 cultures and then as mutant counts from a 10% sample of each culture. Table 1 displays the estimated mean number of mutations per culture and its 95% confidence limits as $\hat{m}$, *m_L_*, and *m_U_* in the case of complete plating, and it displays these same quantities as *m^a^*, *m^a^_L_*, and *m^a^_U_*, respectively, in the case of partial plating. The discrepancies shown in Table 1 indicate that some of the popular methods are not optimal.

**TABLE 1:** Estimates generated by different Web tools^*a*^

Mean no. of mutations per culture					
Web tool	$\hat{m}$	*m_L_*	*m_U_*	*m^a^*	*m_L_^a^*	*m_U_^a^*
FALCOR	9.8237	NA	NA	NA	NA	NA
bz-rates	7.69	NA	NA	30.0	17.05	43.04
FluCalc	9.8237	6.7490	13.3063	38.3975	26.382	52.010
webSalvador	9.8237	6.9522	12.9586	59.0345	45.3258	73.2142

a Shown are the estimated mean number of mutations per culture and 95% confidence limits as *m^^^*, *m_L_* (the lower confidence limit), and *m_U_* (the upper confidence limit) in the case of complete plating, and it displays these same quantities as *m^a^*, *m^a^_L_*, and *m^a^_U_*, respectively, in the case of partial plating. NA, result is not available because either the Web link no longer exists or the Web tool does not offer that result

The first unique feature of webSalvador is its capability to compute likelihood-based confidence intervals (CIs) for mutation rates. Most fluctuation experiments employ a small number of cultures, and this new feature gives improved CIs for small experiments. FALCOR adopted a special kind of CI that is based on the Lea-Coulson formula (5) and that calculates CIs using quantiles of the binomial distribution. When the sample size is small, such CIs tend to have longer lengths and lower coverage rates than likelihood-based CIs.

The second unique feature of webSalvador is that it offers two new approaches to comparing mutation rates. The first approach is likelihood ratio tests specifically tailored for fluctuation assay data (6). These methods are more appropriate for fluctuation assay data because in most investigations, sample sizes are small. The second approach is a statistical estimation method for mutation rate fold change, which to some is more intuitive. A roadblock to the application of this approach has been a lack of methods for constructing CIs for fold change. webSalvador adopts the profile likelihood algorithms developed recently (7).

**FIG 1:**
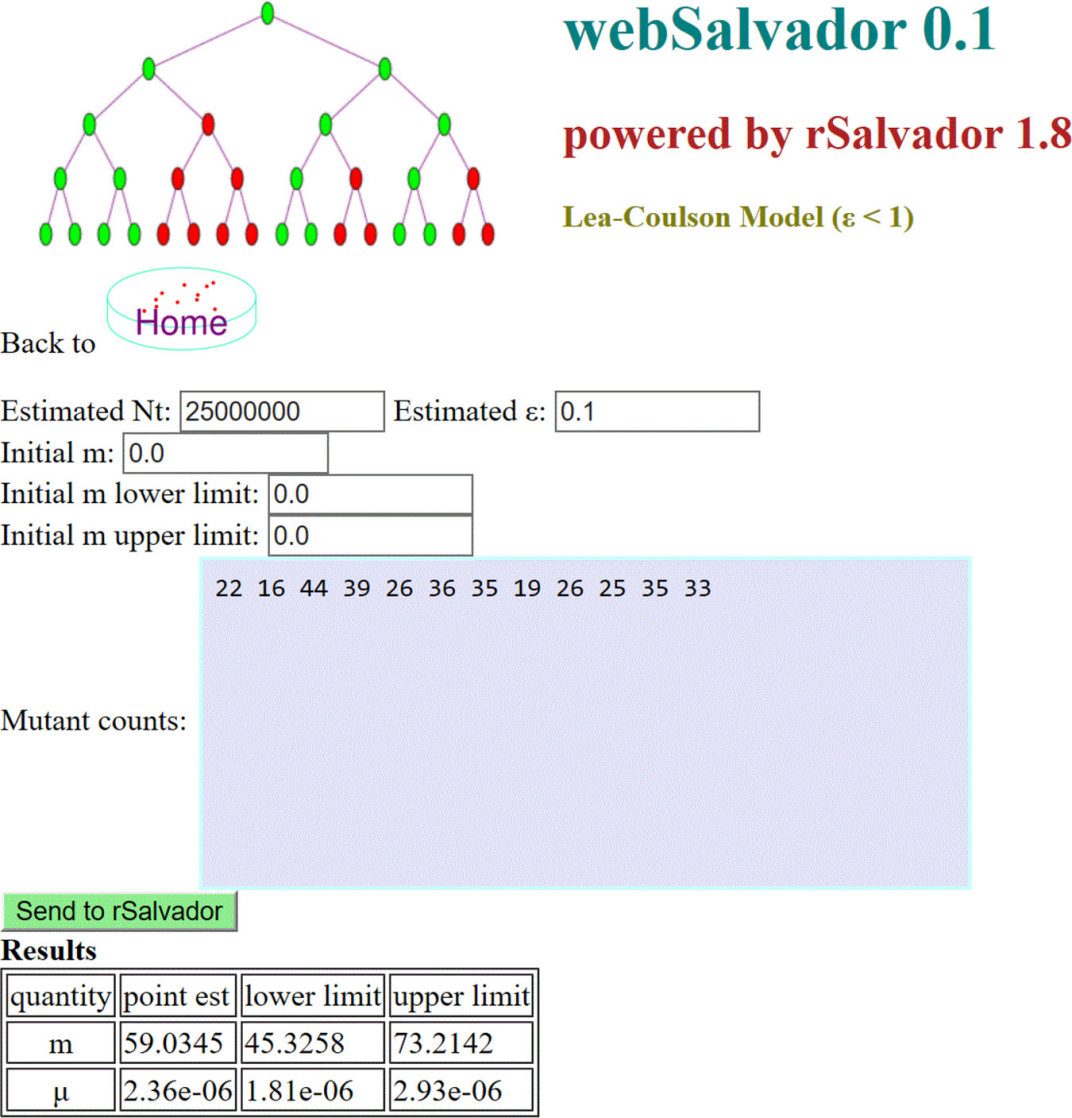
Estimation of both the expected number of mutations, *m*, and the mutation rate, μ, using a modified Lea-Coulson model that accounts for partial plating (here, the plating efficiency is set to 0.1). Note that webSalvador does not adopt the familiar two-column data input format. The two-column format is tedious to use and is unnecessary.

The third unique feature of webSalvador is its likelihood-based approach to accounting for partial plating. Partial plating is sometimes inevitable due to laboratory logistical difficulties. The Stewart correction formula (8) has been widely used in practice, but it can lead to sizable biases (9). webSalvador uses exact algorithms in accounting for partial plating (10) (see Fig. 1).

Among other unique features is the gamma mixture model (11), which can be used to determine whether variation in *N_t_* (the number of cells immediately before plating) is large enough to cause concern.

### Data availability.

The Web tool webSalvador is a user interface to the R package rSalvador (12). Written in the Python language, webSalvador communicates with rSalvador via the Flask Web framework and the rpy2 Python package. webSalvador is accessible at https://websalvador.eeeeeric.com. For a user manual or information on how to host webSalvador locally, visit https://github.com/eeeeeric/rSalvador/tree/master/websalvador.
